# Finding coexisting combinations of posttranslational modifications with HomMTM spectra

**DOI:** 10.1093/bib/bbaf653

**Published:** 2025-12-05

**Authors:** Kunyi Li, Lusheng Wang

**Affiliations:** Department of Computer Science, City University of Hong Kong, 83 Tat Chee Ave., Hong Kong, China; Department of Computer Science, City University of Hong Kong, 83 Tat Chee Ave., Hong Kong, China; City University of Hong Kong Shenzhen Research Institution, Yue Xing Yi Dao, Shenzhen, 518057, China

**Keywords:** coexisting combinations of post-translational modifications, phosphopeptide isoforms identification and quantification, peak error correction alignment

## Abstract

Posttranslational modifications (PTMs) are common biochemical processes that occur after protein synthesis, playing a pivotal role in regulating the activity, localization, stability, and interaction of proteins. Phosphorylation is one of the most crucial and widely studied PTMs, which is widely implicated in cell signal pathways and regulates many cellular processes. For peptide identification with PTMs, tools based on tandem mass spectrum have been developed. However, the existing tools report only one peptide isoform for a given query spectrum, ignoring the probability that multiple isoforms coexist in one single spectrum, which is important for analyzing how different combinations of modifications coexist and compete in cells, leading to diverse functional outcomes. In this paper, we present a workflow to find coexisting combinations of PTMs, aiming at exploring the possibility of simultaneously identifying multiple isoforms from the query spectrum. An algorithm is designed for the identification of at most two isoforms coexisting in a query spectrum. Applying our method to two real phosphopeptide datasets, U2OS and UCEC, we found that coexisting phosphopeptide isoforms occur in 2.16% of the U2OS dataset and 7.19% of the UCEE dataset. To further evaluate the performance of our algorithm for isoform identification, we develop a simulator for generating simulated spectrum with the corresponding ground-truth isoforms. Experiments on the simulated datasets show that our algorithm can achieve an isoform identification accuracy of 85.4%.

## Introduction

Post-translational modifications (PTMs) are common biochemical processes that occur after protein synthesis, playing a pivotal role in regulating the activity, localization, stability, and interaction of proteins. Phosphorylation is one of the most crucial and widely studied PTMs, which is widely implicated in cell signal pathways and regulates many cellular processes. The dysregulation of protein phosphorylation is commonly considered as a hallmark of cancer and autoimmune diseases [1].

Due to the technological development of high-through output mass spectrometry and computational proteomics, several tools for PTM and phospho-peptide identification have been developed, e.g. MaxQuant [2], MS-GF+ [3], MetaMorpheus [4], and MSFragger [5].

As a crucial part in phosphopeptide identification, the localization of phosphorylation sites has been also well-advanced in recent years. Various computational algorithms have been proposed to help determine the confidence level of the potential phosphorylation site candidates in an identified peptide. Mascot delta score [6] and SLIP score [7] determine the phosphorylation site localization based on the identification scores of the identified peptide given by the search engines. Multiple tools assess the confidence of the candidate phosphorylation sites by computing their corresponding probabilities, such as AScore [8], PTM score implemented in MaxQuant [2], pSite [9], etc. Besides, due to the rapid development of artificial intelligence, several algorithms based on deep learning techniques are proposed. DeepRescore2 [10] utilizes the deep learning-based fragment ion intensity prediction and retention time prediction to enhance the localization of phosphorylation site. DeepFLR [11] provides a framework combining the deep learning-based phosphopeptide mass spectrum prediction and a target-decoy approach for false localization rate control in phosphopeptide identification.

However, the existing tools report only one isoform for a given query spectrum, ignoring the probability that multiple isoforms coexist in one single spectrum. Considering the coexistence of multiple candidate phosphorylation sites in one peptide, the lability of phosphate group during peptide fragmentation, and the limited quality of tandem mass spectra when analyzing highly complex samples [12], it is important to explore the chance that the query spectrum corresponds to multiple coexisting phophopeptide isoforms, which are difficult to be separated form the mass spectrum. Such a mass spectrum containing multiple coexisting isoforms is referred to as a *homogeneous multiplexed tandem mass* (HomMTM) spectrum.

Moreover, different phosphorylation combinations (where each combination represents a distinct pattern of phosphorylated or unphosphorylated states across several sites) of a protein coexist and compete in cells, leading to diverse functional outcomes. On the one hand, a single protein can perform different functions depending on which phosphorylation combination dominates [13]. On the other hand, different phosphorylation combinations compete for binding partners, localization, or degradation, creating a dynamic equilibrium [14]. Besides, the abnormal shifts in the distribution of phosphorylation combination have been widely studied and considered relative to the arising of diseases. For instance, Hornbeck *et al*. [15] document disease-associated phosphorylation clusters, and Mertins *et al*. [16] identify co-regulated phosphorylation sites in cancer signaling pathways. Thus, in order to better analyze the phosphoproteomics data and further understand the biological activities resulting from phosphorylation, it is crucial to identify and quantify coexisting phosphopeptide isoforms from the query spectrum.

**Figure 1:**
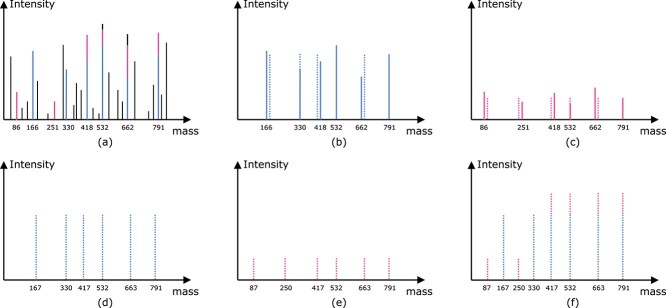
(a) Represents the input mass spectrum where two isoforms coexist, where the blue peaks are from the first isoform, the purple peaks are from the second isoform, the black peaks are the noise peaks. (d) and (e) show the theoretical peaks of the first isoform and the second isoform, respectively. (f) illustrates the theoretical peaks of the spectrum containing the two isoforms. The solid peaks in (b) and (c) are real peaks selected from (a) to fit the theoretical peaks shown in (d) and (e), respectively. The dashed peaks in (b) and (c) are the theoretical peaks of the first and the second isoform, respectively.

**Problem settings:** When multiple isoforms coexist, the input mass spectrum contains three kinds of peaks, peaks from the first isoform, peaks from the second isoform, and noise. See Fig. 1a, where black peaks are noise peaks, blue peaks are from the first isoform, purple peaks are from the second isoform, and some peaks are shared by both isoforms. The theoretical peaks from the first and second isoforms are shown in Fig. 1d and e, respectively. Note that two isoforms may have theoretical peaks with the same mass values and Fig. 1f illustrates the theoretical peaks of the spectrum containing the two isoforms. Our task is to find out the peaks from the input mass spectrum (See Fig. 1a), fitting the two kinds of theoretical peaks in (d) and (e). The solid peaks in Fig. 1b and c are the real peaks selected from the input mass spectrum fitting theoretical peaks of the first and second isoforms, and the dashed peaks are the theoretical peaks from the first and second isoforms, respectively. The intensity errors and mass errors between the real peaks and the theoretical peaks should also be considered here.

In this paper, we present a workflow to find coexisting combinations of PTMs, aiming at exploring the possibility of simultaneously identifying multiple isoforms from the query spectrum, as well as quantifying the abundances of these identified isoforms. An algorithm is designed for the identification for at most two isoforms coexisting in a query spectrum. Applying our method to two real phosphopeptide datasets, U2OS and UCEC, we found that coexisting phosphopeptide isoforms occur in 2.16% of the U2OS dataset and 7.19% of the UCEE dataset. To further evaluate the performance of our algorithm for isoform identification, we develop a simulator for generating simulated spectrum with the corresponding ground-truth isoforms. Experiments on the simulated datasets show that our algorithm can achieve an isoform identification accuracy of 85.4%.

## Materials and Methods

The first step is to use the input mass spectrum to search a protein database and report the corresponding peptide. Based on the reported peptide, we will construct a *peptide isoform mass graph* to represent all possible PTMs as well as all possible isoforms of the peptide. Our computer program will take the mass spectrum and the peptide isoform mass graph as input, try to find all possible *isoform-spectrum matches*, and identify at most two isoforms that can best explain the input mass spectrum.

**Figure 2:**
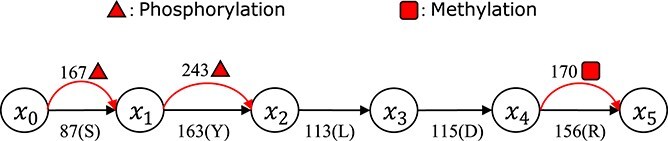
An example of a peptide isoform mass graph with six nodes, representing all possible isoforms for the peptide SYLDR. The directed edge between two nodes represents the original amino acid, whereas the curved edge represents a modification for this specific amino acid. The number on the edge represents the mass of the residue.

**Peptide isoform mass graph:** For a given peptide sequence, the concept of *peptide isoform mass graph* (PMG for short) was proposed in [17] to represent all possible peptide isoforms for the given peptide in a concise way. In a PMG of a peptide with $n$ amino acids, there are $n+1$ nodes organized from left to right in linear order corresponding to the left and right ends of the $n$ amino acids. A black directed edge connecting two neighbor nodes corresponds to an amino acid, with the weight representing the mass of this amino acid. For every potential modification to an amino acid, an additional red edge is added with the modified mass between corresponding nodes. A PMG stores the corresponding normal protein with all its possible isoforms. Figure 2 gives an example.

**Figure 3:**
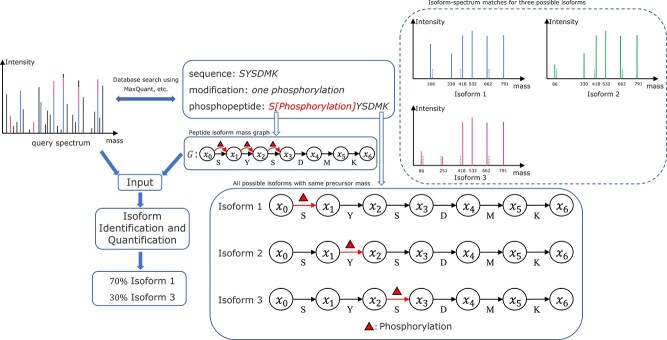
The database search process reports the peptide SYSDMK with one single phosphorylation for the input query mass spectrum. There are three isoforms with the same precursor mass of the input spectrum, where one phosphorylation occurs at the first, second, and third residues of the sequence, respectively. A peptide isoform mass graph $G$ is constructed to represent all possible isoforms based on the reported peptide sequence. Our algorithm finds three isoform-spectrum matches fitting precursor mass of the spectrum using $G$ and the query spectrum. (See the top right corner of the figure.) The solid peaks are real peaks selected from the input query spectrum fitting the corresponding theoretical peaks from the isoforms. The numbers under the peaks are the mass values of the real peaks, while the dashed peaks indicate the theoretical peaks of the isoforms. Finally, two isoform-spectrum matches are selected to best explain the query spectrum and the corresponding abundances are reported as 70% and 30% respectively.

Figure 3 illustrates the workflow using a small example, where the database search process reports the peptide SYSDMK. A peptide isoform mass graph $G$ is constructed based on the peptide SYSDMK to represent all possible modifications and all possible isoforms. There are three isoforms with one phosphorylation at different sites with the same precursor mass of the mass spectrum. The three possible isoform-spectrum matches can be found. Finally, two of them are selected to best explain the input mass spectrum based on the intensity of matched peaks and the corresponding abundances are reported as 70% and 30%, respectively.

A spectrum is formulated as a *spectrum mass graph* (SMG for short). In an SMG, each node corresponds to a unique peak, with the associated mass assigned as the weight. A directed edge is added between a pair of nodes, with its weight as the difference in the weights of the two corresponding nodes.

To simplify the further analysis, we transformed all edges in PMG and SMG into integers through scaling and rounding techniques [18].

The problem of finding isoform-spectrum match is formulated as finding an *alignment* between the two graphs.

**Alignment of PMG and SMG:** Let $x_{0}$, $x_{1}$, $\ldots $, $x_{n}$ be the $n$ nodes in a PMG $G$ and $y_{0}$, $y_{1}$, $\ldots $, $y_{m}$ be the $m$ nodes in an SMG $H$. We use $m_{i}$ to represent the mass value of node/peak $y_{i}$ in $H$. $m_{m} = M_{prec} - Mass(H_{2}O)$, where $M_{prec}$ is the precursor mass of the input mass spectrum. Let $\delta $ be a user-defined number for error tolerance. An alignment of $G$ and $H$ with length $s$ is a sequence of matched pairs $(x_{i_{1}}, y_{j_{1}})$, $(x_{i_{2}}, y_{j_{2}})$, $\ldots $, $(x_{i_{s}}, y_{j_{s}})$, where $0\leq i_{1}<i_{2}<\ldots , i_{s}\leq n$, $0\leq j_{1}<j_{2}<\ldots , j_{s}\leq m$, and there exists a path between node $x_{i_{t}}$ and node $x_{i_{t-1}}$ in $G$ with total mass in the range $[m_{i_{t}}-m_{i_{t-1}}-\delta , m_{i_{t}}-m_{i_{t-1}}+\delta ]$ for $t=2, 3, \ldots , s$.

In practice, we impose the condition that $y_{j_{1}}=y_{0}$ and $y_{j_{s}}=y_{m}$ to match the whole mass spectrum to a segment in $G$.

The above alignment definition imposes that the mass difference between two consecutive matched peaks is roughly the same (with error tolerance value $\delta $) as the mass of the corresponding sub-path in $G$. This method may suffer from error accumulation, where $k$ consecutive positive/negative errors in the alignment will lead to an error of $k\delta $ between a pair of peaks in $H$. This is certainly evidence that such an alignment is not reliable.

To get more realistic alignments, Zhan and Wang [19] proposed a model considering peak error correction.

**Peak error correction alignment:** A length $r$ peak error correction alignment for a PMG $G$ and SMG $H$ is a sequence of triples $(x_{i_{1}}, y_{j_{1}}, k_{j_{1}})$, $(x_{i_{2}}, y_{j_{2}}, k_{j_{2}})$, $\cdots $, $(x_{i_{r}}, y_{j_{r}}, k_{j_{r}})$, where $k_{j_{q}}\in (-\delta , \delta )$ is the error correction value for peak $y_{j_{q}}$ such that $m_{j_{q}} + k_{j_{q}} - (m_{j_{q-1}} + k_{j_{q-1}}) = M_{i_{q-1},i_{q}}$, where $m_{j_{q}}$ is the mass for the peak $y_{j_{q}}$ and $M_{i_{q-1},i_{q}}$ is the mass of a path between node $x_{i_{q-1}}$ and node $x_{i_{q}}$. Note that $\delta $ represents the error tolerance value for the mass of a peak. The *error correction alignment problem* is to find a error correction alignment with the maximum alignment size between $G$ and $H$ [19]. Suppose an SMG $H$ containing the six real peaks $y_{1}, y_{2},..., y_{6}$ shown in Fig. 1b, where $m_{1} = 166, m_{2}=330, m_{3}=418, m_{4}=532, m_{5}=662, m_{6}=791$. Let $G$ represent the phosphopeptide isoform 1 in Fig. 3; the error correction alignment between $H$ and $G$ is $(x_{1},y_{1},1)(x_{2},y_{2},0)(x_{3},y_{3},-1)(x_{4},y_{4},0)(x_{5},y_{5},1)(x_{6},y_{6},0)$.

### Finding all error correction alignments of $H$ and $G$

Let $x_{0}$, $x_{1}$, $\ldots $, $x_{n}$ be the $n$ nodes in a PMG $G$ and $y_{0}$, $y_{1}$, $\ldots $, $y_{m}$ be the $m$ nodes in an SMG $H$. Here we are not only interested in alignments with the largest size. We are interested in error correction alignments starting with $(x_{0}, y_{0}, 0)$ and ending with $(x_{n}, y_{m^{\prime}}, k)$ for some $k\leq \delta $ such that $m_{m^{\prime}}+k = M$, $M = MolecularMass - Mass(H_{2}O)$, where $MolecularMass$ is the molecular mass of the isoform corresponding to $G$. We do not want the largest size alignments here, since we want to consider some isoforms/alignments, where a few peaks in the spectrum are missing.

**Figure 4:**
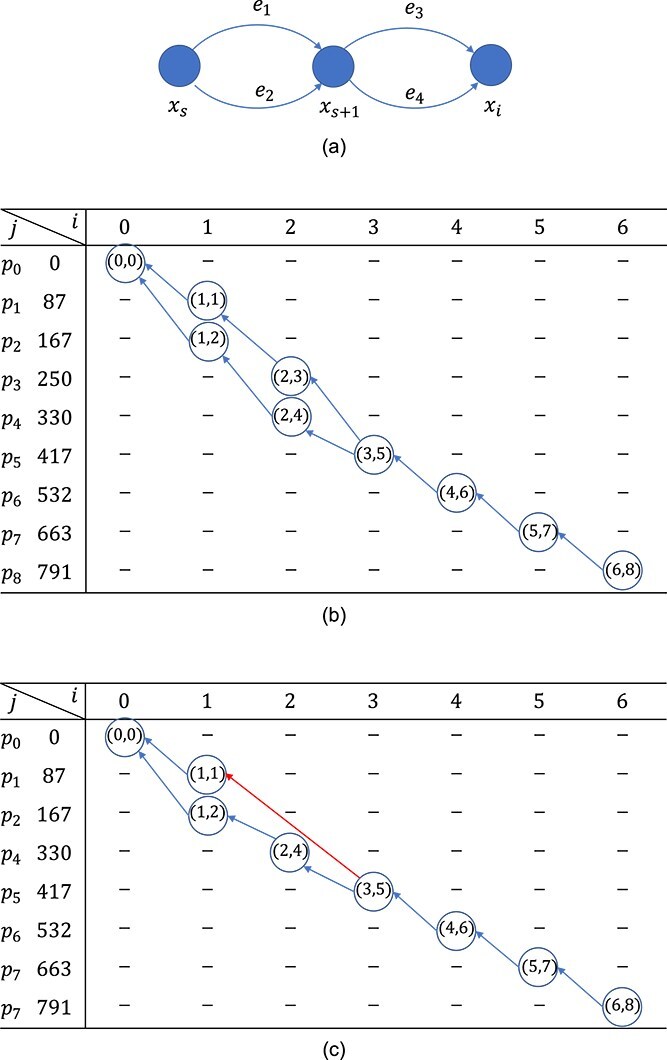
(a) An example of paths from $x_{s}$ to $x_{i}$. (b) An example of a backtracking graph. (c) An example of a backtracking graph when a peak is missing.

Let $d(s,i)$ denote the set of distinct path masses from node $x_{s}$ to node $x_{i}$ in $G$. Each mass $m \in d(s,i)$ corresponds to a set $P(s, i, m)$ of paths from node $x_{s}$ to node $x_{i}$ in $G$. For example, $x_{s}$ and $x_{i}$ are shown in Fig. 4a. The mass $m(e_{i})$ is the mass on edge $e_{i}$, where $m(e_{1})=m(e_{3})=1$ and $m(e_{2})=m(e_{4})=2$. Then we have $d(s, i)=\{2, 3, 4\}$, $P(s, i, 2)=\{e_{1}e_{2}\}$, $P(s, i, 3)=\{e_{1}e_{4}, e_{2}e_{3}\}$, $P(s, i, 4)=\{e_{2}e_{4}\}$.

Let $(x_{i}, y_{j}, k)$ be an element of an error correction alignment, where peak $y_{j}$ in $H$ is matched to node $x_{i}$ in $G$ with error correction value $k$. We define $E(i,j,k)$ to be the set of triples $(i^{\prime}, j^{\prime}, k^{\prime})$ such that there exists an $m\in d(i^{\prime}, i)$ such that 


(1) \begin{eqnarray*} ~m-[(m_{j}+k)-m_{j^{\prime}}]=k^{\prime}. \end{eqnarray*}

**Constructing backtracking graph** In order to find all isoforms that fit the input mass spectrum, we constructed a graph during the backtracking process. Note that we are interested in alignments ending with $y_{m}$, where $m_{m}+k$ is equal to $M = MolecularMass - Mass(Water)$ after error correction, where $MolecularMass$ is the molecular mass of the isoform. Thus, we set ${\mathcal E}=\{E(n, m, k)\}$. Note that the value of $k$ is fixed by $M=m_{m}+k$ and ${\mathcal E}$ contains only one node.

Let $Q$ be a quque. The backtracking graph $B$ is constructed as in Algorithm 1:


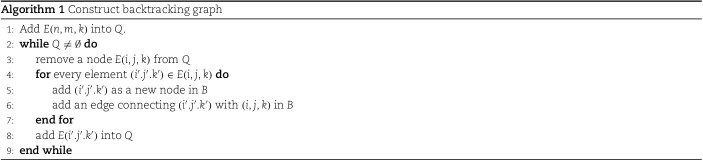


For any error correction alignment starting from $(0, 0, 0)$ and ending at a node $u\in{\mathcal E}$, there is a corresponding path in the backtracking graph $B$. In other words, $B$ contains all the alignment starting from $(0, 0, 0)$ and ending at a node in ${\mathcal E}$.

Example 1. To illustrate the backtracking graph construction, we use a small example. Let $H$ be a spectrum containing $p_{0}$, $p_{1}$, $\ldots $, $p_{8}$ peaks with the corresponding masses $m_{0}=0$, $m_{1}=87$, $m_{2}=167$, $m_{3}=250$, $m_{4}=330$, $m_{5}=417$, $m_{6}=532$, $m_{7}=663$, and $m_{8}=791$. The nine peaks form the two isoforms given in Fig. 1d and e, respectively. For simplicity, we assume that the peaks have no error, i.e. $\delta =0$. The protein mass graph $G$ for peptide sequence $SYSDMK$ contains seven nodes. The matrix $E(i,j)$ with $\delta =0$ obtained from the dynamic programming algorithm is given in Fig. 4b.

The backtracking graph is constructed from $E(6, 8)$ and we have ${\mathcal E}=\{E(6, 8)\}$. $E(6,8)=\{(5, 7)\}$, $E(5, 7)=\{(4, 6)\}$, $E(4, 6)=\{(3, 5)\}$, $E(3,5)=\{(2,3), (2,4)\}$, $E(2,3)=\{(1,1)\}$, $E(2,4)=\{(1,2)\}$, $E(1,1)=E(1,2)=\{(0,0)\}$. The backtracking graph contains nine blue nodes and nine blue directed edges.

Our method also considers the isoforms/alignments with a few missing peaks.

Example 2. If peak $p_{3}$ in Example 1 is missing, we will have a backtracking graph as shown in Fig. 4c. The backtracking graph is constructed from $E(6, 8)$, where ${\mathcal E}=\{E(6, 8)\}$. Based on Algorithm 1, we have $E(6,8)=\{(5, 7)\}$, $E(5,7)=\{(4, 6)\}$, $E(4, 6)=\{(3, 5)\}$, $E(3,5)=\{(2,4),(1,1)\}$, $E(2,4) = \{(1,2)\}$, and $E(1,1) = E(1,2) = \{(0,0)\}$. The backtracking graph contains eight colored nodes, seven blue directed edges, and one red directed edge.

Note that the backtracking graph contains all possible alignments corresponding to the candidate isoforms for the input spectrum. In general, the number of possible isoforms in the backtracking graph is very large and we need to have a way to find the two (we assume that there are at most two isoforms for each input spectrum) real isoforms and their corresponding abundance. The algorithm for this step is given in the next subsection, where we basically want to find two paths in the backtracking graph such that the intensity error of corresponding peaks is minimized.

### Isoform identification

When a mass spectrum contains multiple isoforms, we want to find the isoforms and their abundances. The intensities of matched peaks in the corresponding alignments can be used for finding isoforms and their abundances [20]. Here we consider the case, where there are two isoforms.

Let $A=(x_{i_{1}}, y_{j_{1}}, k_{j_{1}})$, $(x_{i_{2}}, y_{j_{2}}, k_{j_{2}})$, $\cdots $, $(x_{i_{r}}, y_{j_{r}}, k_{j_{r}})$ be the error correction alignment for an isoform $P$. Let $q$ be the theoretical peak intensity for the isoform. The total intensity error for the isoform is defined as $e(A)=\sum _{t=1}^{r} |q-I_{j_{t}}|$, where $I_{j_{t}}$ is the intensity of peak $y_{j_{t}}$.

**Problem formulation:** Let $A_{1}$ and $A_{2}$ be two alignments/paths in $B$. A pair of two paths $A1$ and $A2$ is *valid* if both paths start with $E(0,0,0)$ and end at $E(n, m, k)\in{\mathcal E}$. The *isoform identification and quantification* problem is to find a pair of valid alignments/paths $A1$ and $A2$ from $B$ and the corresponding theoretical intensities $q_{1}$ and $q_{2}$ such that $e(A_{1})+e(A_{2})$ is minimized. The two isoforms corresponding to the two alignments found in this way is considered to best explain the input mass spectrum.

The abundance of the isoform corresponding to $A_{i}$ is $\frac{q_{i}}{q_{1}+q_{2}}$ for $i=1$ and $2$. Note that $q_{1}$ and $q_{2}$ are not known. Let $I_{max}$ be the maximum peak intensity for all the peaks involved in $B$. We can guess the values of both $q_{1}$ and $q_{2}$ as $I_{max}\times x\%$, where $x=1, 2, \ldots , 100$.

**Intensity error of two paths:** Let us consider a valid pair of paths $P_{1}=E(0,0,0)E(i_{1}, j_{1}, k_{1})\ldots $  $E(i_{q}, j_{q}, k_{q})$ and $P_{2}=E(0,0,0) E(i^{\prime}_{1}, j^{\prime}_{1}, k^{\prime}_{1})\ldots $  $E(i^{\prime}_{q^{\prime}}, j^{\prime}_{q^{\prime}}, k^{\prime}_{q^{\prime}})$ in $B$. Since the two isoforms may share some common peaks, we have to define the intensity error of a shared peak. Let $u=E(i, j, k)$ and $v=E(i^{\prime}, j^{\prime}, k^{\prime})$ be two nodes in $B$ with $j>0$ and $j^{\prime}>0$. Let $I(u)$ be the intensity value of peak $p_{j}$ of node $u$. The intensity error for a shared peak is defined as follows: 


(2) \begin{eqnarray*} \varepsilon(u, v) = |I(u) - q_{1} - q_{2}| & \textrm{if}\ j = j^{\prime} ~\&~ k=k^{\prime}. \end{eqnarray*}

Since each peak has only one correct position, we do not consider the case, where $j=j^{\prime}$ and $k\neq k^{\prime}$.

Let $u=E(i, j, k)$ be a node in $B$. When peak $p_{j}$ is not shared, we denote $\varepsilon (u)_{1}$ and $\varepsilon (u)_{2}$ be the intensity errors of peak $p_{j}$ as in the first and second paths, respectively. 


(3) \begin{align*} \varepsilon(u)_{1}=|I(u)-q_{1}|;\end{align*}

(4) \begin{align*} \varepsilon(u)_{2}=|I(u)-q_{2}|. \end{align*}

Let $S(P_{1}, P_{2})$ be the set of pairs of nodes $u=E(i, j, k)$ and $v=E(i^{\prime}, j, k)$, where $u$ and $v$ share a common peak $p_{j}$. Let $P_{i}^{n}$ be the set of nodes $E(i, j, k)$ in $p_{i}$ for $i=1,2$ that do not share peak $p_{j}$ with any node in the other path. The total peak intensity error $I(P_{1}, P_{2})$ of both $P_{1}$ and $P_{2}$ can be computed as follows: 


(5) \begin{eqnarray*} I(P_{1}, P_{2})=\sum_{(u, v)\in S(P_{1}, P_{2})} \varepsilon(u, v)+\sum_{u\in P_{1}^{n}} \varepsilon(u)_{1} +\sum_{v\in P_{2}^{n}} \varepsilon(v)_{2}. \end{eqnarray*}

Note that, if a peak is missing in an alignment/path in $B$, we add the missing peak at its theoretical position with intensity value $0$. (See Example 4.)

Example 3. Let $I_{j}$ be the intensity values of $p_{j}$ in example 1, where $I_{0}=0$, $I_{1}=39$, $I_{2}=75$, $I_{3}=33$, $I_{4}=76$, $I_{5}=105$, $I_{6}=110$,$I_{7}=92$, $I_{8} = 99$. Let $q_{1}=70$ and $q_{2}=30$. The intensity error for the two peptide isoforms is $|99-100| + |92-100| + |110-100|+|105-100|+|76-70|+|33-30|+|75-70|+|39-30|$.

We need to consider the case, where some peaks in the isoforms are missing.

Example 4. When peak $p_{3}$ is missing in Example 3, the backtracking graph is shown in Fig. 4c, where the two ends of the red edge from a pair of peaks, and there is a missing peak in the middle. In this case, the intensity error becomes $|99-100| + |92-100| + |110-100|+|105-100|+|76-70|+|0-30|+|75-70|+|39-30|$.

The isoform identification and quantification problem can be formally defined as finding two valid paths in $B$ with minimum intensity error.

**The algorithm:** Now, we present an algorithm to find two valid paths in $B$ with minimum intensity error. It is worth to point out that a peak may match different nodes in the protein mass graph (since it is possible that two paths starting from $x_{0}$ and ending at different nodes may have the same total mass). This case needs to be carefully considered in algorithm design.

We consider two sub-paths heading at nodes $u =E(i,j,k)$ and $v=E(i^{\prime},j^{\prime},k^{\prime})$ to the right ends in ${\mathcal E}$ for valid pairs of paths in $B$. Let $D(u, v)$ be the minimum intensity errors for the two sub-paths heading at $u$ and $v$.

When $j=j^{\prime}$ and $k=k^{\prime}$, we can compute $D(u, v)$ as follows: 


(6) \begin{eqnarray*}\nonumber D(u, v) = \varepsilon(u, v) +d(u, u^{\prime},1)+d(v, v^{\prime},2)\\ +\min_{u^{\prime}\in f(u), v^{\prime}\in f(v)} D(u^{\prime}, v^{\prime}), \end{eqnarray*}

where $f(v)$ is the set of nodes $v^{\prime}$ such that there is an edge $(v^{\prime}, v)$ in $B$, and $d(u, u^{\prime}, 1) =n_{1}\times q_{1}$ and $d(v,v^{\prime},2)=n_{2}\times q_{2}$ are the intensity error for the $n_{1}$ and $n_{2}$ missing peaks on edges $(u^{\prime}, u)$ and $(v^{\prime}, v)$, respectively.

When $j\neq j^{\prime}$, we can compute $D(u, v)$ as follows.

If $j<j^{\prime}$, 


(7) \begin{eqnarray*} D(u, v) = \varepsilon(u)_{1} +d(u, u^{\prime},1)+\min_{u^{\prime}\in f(u)} D(u^{\prime}, v). \end{eqnarray*}

If $j>j^{\prime}$, 


(8) \begin{eqnarray*} D(u, v) = \varepsilon(v)_{2} +d(v, v^{\prime},2)+\min_{v^{\prime}\in f(v)} D(u, v^{\prime}). \end{eqnarray*}

Example 5. Let $u=E(2,4)$ and $v = E(1,1)$ in Example 2; we can take the computation of $D(E(2,4),E(1,1))$ with the intensities mentioned in Example 3 as an example. In this case, $j = 4> j^{\prime} = 1$. Thus, $D(u,v)$ is computed following the equation (8). $\varepsilon (v)_{2} = |I_{1} - q_{2}| = |39-30| = 9$. Based on the backtracking graph shown in Fig. 4c, $f(v) = f(E(1,1)) = \{E(3,5)\}$. Since $f(v)$ only contains one node, $v^{\prime} = E(3,5)$, $min_{v^{\prime}\in f(v)} D(u, v^{\prime}) = D(E(2,4),E(3,5))$. One peak $p_{3}$ is missing on edge from $v^{\prime}=E(3,5)$ to $v=E(1,1)$, thus $d(v,v^{\prime},2) = n_{2}\times q_{2} = 1 \times 30 = 30$. Therefore, $D(E(2,4),E(1,1)) = \varepsilon (E(1,1))_{2} + d(E(1,1), E(3,5),2) + D(E(2,4),E(3,5)) = 9 + 30 + D(E(2,4),E(3,5))$.

**Figure 5:**
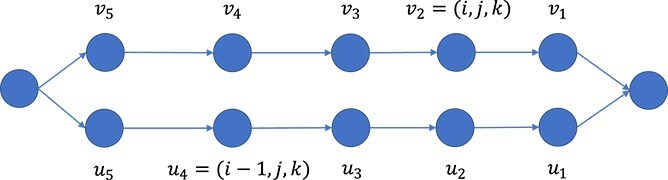
The same peak $p_{j}$ appears in both paths.

The reason that we use equations (6) is to make sure that the intensity error of the peak that appears in both isoforms/paths can be computed correctly using equation (2). Let us consider the two paths in Fig. 5, where $E(i, j, k)$ and $E(i-1, j, k)$ appear in both paths. Thus, the intensity error of peak $p_{j}$ should be computed using equation (2). If we compute $D(u_{i}, v_{i})$ for $i=1,2, \ldots , 5$ with the increasing order of $i$, the intensity error of peak $p_{j}$ cannot be computed correctly.

Finally, $D((0, 0, 0), (0, 0, 0))$ contains the minimum intensity error of two valid paths in $B$. A standard backtracking process can report the corresponding two valid paths in $B$. Apparently, we do not need to compute $D(u, v)$ for all pairs of $(u, v)$ in $B$. For example, there is no need to compute $D((i, 0, k), (i^{\prime}, m, k^{\prime}))$ since $p_{0}$ is the first peak and $p_{m}$ is the last peak in the spectrum.

Now, we design a way to compute all the required $D(u, v)$s. Here we need to use a queue $Q_{1}$ and a list $Q_{2}$. First, we add the pair $(((0, 0, 0), (0, 0, 0))$ into $Q_{1}$. When $Q_{1}$ is not empty, we remove an element $((i,j,k), (i^{\prime},j^{\prime}, k^{\prime}))$ from $Q_{1}$ and add it to $Q_{2}$. If none of the two nodes is in ${\mathcal E}$, two cases arise:

Case 1: $j=j^{\prime}$. Add all the pairs $(u, v)$, where $u\in f((i,j,k))$ and $v\in f((i^{\prime},j^{\prime},k^{\prime})$, to $Q_{1}$. This case includes the sub-case, where $i=i^{\prime}$.

Case 2: $j\neq j^{\prime}$. If $j>j^{\prime}$, then add $((i,j,k), v)$, where $v\in f((i^{\prime},j^{\prime},k^{\prime})$, to $Q_{1}$. If $j<j^{\prime}$, then add $(u, (i^{\prime},j^{\prime},k^{\prime}))$, where $u\in f((i,j,k))$, to $Q_{1}$.

If $(i,j,k)$ is in ${\mathcal E}$ and $(i^{\prime},j^{\prime},k^{\prime})\not \in{\mathcal E}$, we add $(u, (i^{\prime},j^{\prime}, j^{\prime}))$ to $Q_{1}$, where $u\in f((i,j,k))$.

If $(i^{\prime},j^{\prime},k^{\prime})$ is in ${\mathcal E}$ and $(i,j,k)\not \in{\mathcal E}$, we add $((i,j, j), v)$ to $Q_{1}$, where $v\in f((i^{\prime},j^{\prime},k^{\prime}))$.

if both $(i,j,k)$ and $(i^{\prime},j^{\prime}, k^{\prime})$ are in ${\mathcal E}$, we do not add anything to $Q_{1}$.

The above process will be repeated until $Q_{1}$ becomes empty. At the end, $Q_{2}$ contains all pairs of $(u, v)$ used to compute $D(u, v)$. We can compute $D(u, v)$ using equations (6)-(8) in the inverse order of $Q_{2}$

The algorithm is described as Algorithm 2.


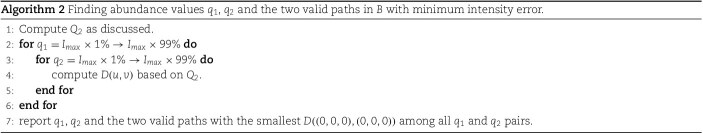


## Results

### Real datasets

U2OS dataset and UCEC dataset are the two real-world LC-MS/MS phosphoproteome datasets. Here we use them for phosphopeptide isoform identification and quantification. U2OS dataset is a label-free phospho-proteome dataset generated from *U2OS* cell line, which can be downloaded from the PRIDE database with the accession key PXD023665 [21]. UCEC dataset is a TMT10-labeled phosphoproteome dataset from the National Cancer Institute’s Clinical Proteomic Tumor Analysis Consortium (CPTAC) uterine corpus endometrial carcinoma (*UCEC*) study with the accession key PDC000126 [22].

The spectra of the two LC-MS/MS phosphoproteome datasets are first searched against the human protein database (Uniprot:v20190214) by Yi *et al*. [10] using four different phosphopeptide identification tools (Comet [23], MaxQuant, MS-GF+ and XTandem [24]) to get a set of peptide-spectrum matches(PSMs). During the search, oxidation of methionine (UNIMOD Accession number: 35) and phosphorylation of serine, threonine, and tyrosine (UNIMOD Accession number: 21) are considered as variable modifications. The identified PSMs are filtered to a 1% FDR(False Discovery Rate) at both PSM and peptide levels. In this way, 37 595 PSMs and 52 863 PSMs are extracted for U2OS dataset and UCEC dataset, respectively. Our algorithm is utilized to conduct coexisting isoform identification and quantification for each of the 37 595+52 863 extracted PSM, where oxidation and phosphorylation are used as variable modifications (following the modification settings of the database search step in [10]). The results are shown in Table 1. Two coexisting isoforms are identified for 811 PSMs obtained from U2OS dataset and 3800 PSMs obtained from UCEC dataset, respectively. For convenience, the PSMs where two coexisting isoforms are identified are refered to as *HomMTM PSMs*.

**Table 1:** HomMTM identification results

Dataset	Total PSM	HomMTM PSM	Percentage
U2OS	37 595	811	2.16%
UCEC	52 863	3800	7.19%

**Figure 6:**
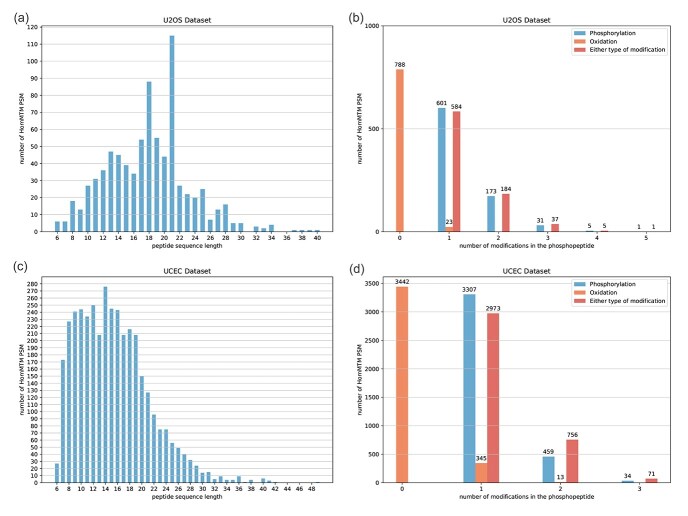
(a) and (c) show the peptide sequence length distributions of HomMTM PSMs obtained from U2OS dataset and UCEC dataset, respectively. (b) and (d) show the modification distributions of HomMTM PSMs obtained from U2OS dataset and UCEC dataset, respectively. The blue/orange bars in (b) and (d) indicate the number of HomMTM PSMs where specific numbers of phosphorylations/oxidations are found in the identified peptide isoforms. The red bars indicate the number of HomMTM where specific numbers of either phosphorylations or oxidations are found in the indentified peptide isoforms.

The peptide length distributions of HomMTM PSMs obtained from U2OS dataset and UCEC dataset are shown in Fig. 6a and c, respectively. The modification distributions of HomMTM PSMs obtained from U2OS dataset and UCEC dataset are shown in Fig. 6b and d, respectively. Phosphorylation occurrs at least once on peptides for all of the HomMTM PSMs, while oxidation rarely occurs more than once. For the majority of HomMTM PSMs, phosphorylation occurs only once on the peptides.

We divide the HomMTM PSMs into three types according to the different modification site between two identified isofroms:

- Isoforms are only different in phosphorylation site;
- Isoforms are only different in oxidation site;
- Isoforms are different in both phosphorylation site and oxidation site.

The numbers of different types of HomMTM PSMs for U2OS dataset and UCEC dataset are shown in Table 2. Since phosphorylation can occur on three types of amino acid residues (serine, threonine, and tyrosine), isoforms different in phosphorylation site are identified in most of the HomMTM PSMs. For U2OS dataset, among 810 HomMTM PSMs where the isoforms are different in phosphorylation site, one different phosporylation site between the identified isoforms is reported for 805 cases, while two different phosporylation sites between the identified isoforms are reported for the remaining five cases. For UCEC dataset, among 3792 HomMTM PSMs, one different phosphorylation site and two different phosphorylation sites are reported for 3769 cases and 23 cases, respectively.

**Table 2:** Numbers of different types of HomMTM PSM

Dataset	Type	Number
U2OS	only different in phosphorylation site	810
	only different in oxidation site	1
	different in both phosphorylation site and oxidation site	0
UCEC	only different in phosphorylation site	3788
	only different in oxidation site	8
	different in both phosphorylation site and oxidation site	4

Some special cases of HomMTM PSMs are displayed in Table 3:

- Case 1: five phosphorylations are reported to occur on the peptide with one different phophorylation site between two isoforms;
- Case 2: two phosphorylations are reported to occur on the peptide, where both two phosphorylation sites are different between two isoforms;
- Case 3: one phosphorylation and one oxidation are reported to occur on the peptide, where two isoforms are only different in oxidation site;
- Case 4: one phosphorylation and one oxidation are reported to occur on the peptide, where both phosphorylation site and oxidation site are different between two isoforms.

**Table 3:** Special HomMTM cases

	Isoform	Abundance
Case 1	$KKAS^{\textrm{a}}S^{\textrm{a}}SDS^{\textrm{a}}EDS^{\textrm{a}}S^{\textrm{a}}EEEEEVQGPPAK$	18.2%
	$KKASS^{\textrm{a}}S^{\textrm{a}}DS^{\textrm{a}}EDS^{\textrm{a}}S^{\textrm{a}}EEEEEVQGPPAK$	81.8%
Case 2	$SYLEGS^{\textrm{a}}S^{\textrm{a}}DNQLKDSESTPVDDR$	37.5%
	$S^{\textrm{a}}Y^{\textrm{a}}LEGSSDNQLKDSESTPVDDR$	62.5%
Case 3	$VSEEQTQPPS^{\textrm{a}}PAGAGM^{\textrm{b}}STAMGR$	27.3%
	$VSEEQTQPPS^{\textrm{a}}PAGAGMSTAM^{\textrm{b}}GR$	72.7%
Case 4	$HLSS^{\textrm{a}}EEMM^{\textrm{b}}R$	21.4%
	$HLS^{\textrm{a}}SEEM^{\textrm{b}}MR$	78.6%

$^{\textrm{a}}$ Phosphorylation. $^{\textrm{b}}$Oxidation

**Enriched phosphoproteomics dataset:** Now we try to further evaluate the performance of our method in coexisting isoforms identification using a enriched phosphoproteomics dataset of bottom-up phosphopepide spectra constructed by Chang *et al*. [25], which can be downloaded from the PRIDE database with the accession key PXD041271. The spectra are searched against the human protein database by Chang *et al*. using Comet [23] to get a set of phophorylation PSMs. During the search, N-term acetylation (UNIMOD Accession number: 1), Oxidation of methionine (UNIMOD Accession number: 35), and phosphorylation of serine, threonine, and tyrosine (UNIMOD Accession number: 21) are considered as variable modifications. The identified PSMs are filtered to a 1% FDR at PSM level. In this way, 19 606 PSMs are obtained for coexisting isoform identification and quantification.

Considering the fact that the peaks we used to construct the alignment between SMG and PMG are potential b-ions and y-ions, the number of b-ions and y-ions corresponding to the residues of the peptide in the query spectrum strongly affects the performance of our method. Given a PSM where a peptide $P$ matches a query spectrum $S$, the number of residues in the peptide is $n$, and the number of peptide bonds is $n-1$. For a peptide bond $B$ in $P$, if there exists a peak in $S$ whose mass is equal to the theoretical mass of b-ion or y-ion generated from the cleavage of $B$ with a tolerance (0.1 Dalton is used in our method), we say that $B$ is supported by a peak in $S$. Let $m$ be the number of peptide bonds that are supported, we denote the *peak-support rate* by $r=\frac{m}{n-1}$ for the PSM.

The 19 606 PSMs are divided into four groups with different ranges of peak-support rates $(0\%,25\%],(25\%,50\%],(50\%,75\%], (75\%,100\%]$, respectively. The results of coexisting isoform identification of PSMs in each group are displayed in Table 4. We can see that the percentage of HomMTM PSMs in the group with the highest peak-support rate is considerably higher than that in the rest of the groups, and the low peak-support rate ($<50\%$) makes our method hardly identify two different isoforms from the PSM.

**Table 4:** Results of isoforms identification on enriched phosphoproteomics dataset

Peak-support rate $r$	$r\in (0\%,25\%]$	$r\in (25\%,50\%]$	$r\in (50\%,75\%]$	$r\in (75\%,100\%]$
Number of total PSMs	1024	4860	6057	7665
Number of HomMTM PSMs	0	1	57	346
Percentage of HomMTM PSMs	0%	0.02%	0.94%	4.51%

**Figure 7:**
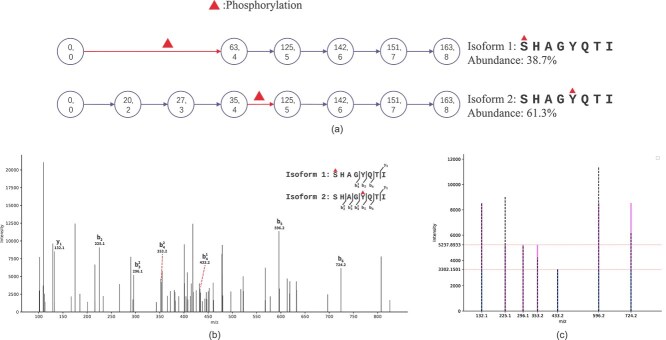
An example of a HomMTM PSM where one of the identified isoforms is identical to the isoform reported by Comet. For the input PSM, the query spectrum can be found with scans 7507 in the raw file with the number 35765 in the enriched phosphoproteomics dataset, and the corresponding peptide reported by Comet is $SHAGYQTI$ with a phophorylation occurring at the fifth amino acid residue. Besides the isoform reported by Comet, our method identifies another isoform with a phophorylation occurring at the first amino acid residue coexisting in the query spectrum. (a) is a graphical illustration of the isoforms identification and quantification results for the input PSM. The two numbers on each nodes represent the peak number and node number in the alignment, respectively. In this case, two isoforms with one different phosphorylation site are identified, where isoform 2 is identical to the identification result of Comet. The peak supposed to match the first node is missing for both the isoforms. Besides, the peaks supposed to match the second and third nodes of the isoform 1 are missing in the spectrum. (b) displays the query spectrum with annotated peaks corresponding to the identified isoforms. Peaks $\mathrm{b}_{5}, \mathrm{b}_{6}, \mathrm{y}_{1}$ are shared by two isoforms. $\mathrm{b}^{1}_{4}$ represents the peak only corresponding to the $\mathrm{b}_{4}$ ion in isoform 1, while $\mathrm{b}^{2}_{2}, \mathrm{b}^{2}_{3}, \mathrm{y}^{2}_{4}$ are peaks only corresponding to isoform 2. (c) displays the spectrum containing the real peaks corresponding to the two identified isoforms and the artificial peaks with theoretical intensities of two isoforms computed by our method. Specifically, the black dashed lines represent the real peaks in (b). The blue lines and the purple lines represent the artificial peaks corresponding to isoform 1 and isoform 2, respectively, where the heights of the blue peaks and the purple peaks indicate the computed theoretical intensities of the two identified isoforms. The height difference between the solid line and the vertical dashed line at the same horizon position indicates the intensity error of the real peak.

Now we try to analyze the identification results of our method by comparing the isoforms identified by our method and the isoforms identified by Comet. Two types of PSMs appear in the results of the coexisting isoform identification: HomMTM PSMs and normal PSMs where only one isoform is identified. For HomMTM PSMs, if one of the two isoforms identified by our method is identical to the isoform reported by Comet, the identification is considered *common identification*. Figure 7 shows an example of a HomMTM where the second identified isoform is identical to the isoform reported by Comet. For normal PSMs where only one isoform is identified, the identification is a common identification if the identified isoform reported by our method is identical to the isoform repoeted by Comet. The percentage of common identifications for the two types of PSMs in different groups are displayed in Tables 5 and 6, respectively. We can see that for PSMs in the groups with the peak-support rates lower than 50%, the the percentages of common identifications are low. For PSMs in the groups with higher peak-support rates, the percentages of common identifications increases to a more substantial level. Considering the fact that our method only utilizes the potential b-ions and y-ions in the query spectrum to identify isoforms, the difference between the identification results of our method and the classical peptide search method, such as Comet [23], is foreseeable. Thus, the identification results on the enriched phosphoproteomics dataset indicates an acceptable performance of our method.

**Table 5:** Percentage of common identification of HomMTM PSMs

Peak-support rate $r$	$r\in (0\%,25\%]$	$r\in (25\%,50\%]$	$r\in (50\%,75\%]$	$r\in (75\%,100\%]$
Number of HomMTM PSMs	0	1	57	346
Percentage of common identifications	-	0%	66.7%	85.0%

**Table 6:** Percentage of common identifications of PSMs where only one isoform is identified

Peak-support rate $r$	$r\in (0\%,25\%]$	$r\in (25\%,50\%]$	$r\in (50\%,75\%]$	$r\in (75\%,100\%]$
Number of normal PSMs	1024	4859	6000	7319
Percentage of common identifications	37.7%	49.6%	63.3%	69.0%

### Simulated dataset

**HomMTM spectrum simulator:** To further evaluate the peptide isoforms identification performance of our method, we design a pipeline to generate simulated HomMTM spectra based on the real HomMTM PSMs reported in the previous experiment for U2OS dataset and UCEC dataset.

For an input HomMTM PSM reported for a real spectrum $S$, let $F_{1}$, $F_{2}$ be the two different identified isoforms, the reported theoretical intensities of the peaks corresponding to $F_{1}$ and $F_{2}$ in $S$ are $q_{1}, q_{2}$, respectively. To generate a simulated HomMTM spectrum $S^{\prime}$ based on the input HomMTM PSM, the simulation is divided into two steps: generating sound peaks and generating noise peaks.

*Generating sound peaks* The simulator first generates two lists $S^{*}_{1}$ and $S^{*}_{2}$, which contain the masses of peaks corresponding to the theoretical ions of amino acids in $F_{1}$ and $F_{2}$, respectively. Take generating $S^{*}_{1}$ as an example; for each amino acid in $F_{1}$, a mass of the peak corresponding to the theoretical ion is generated. After the masses corresponding to the theoretical ions of all amino acids in $F_{1}$ are generated, some masses are randomly abandoned with *the missing peak probability*, which is a parameter of the simulator used to specify the probability that a sound peak is missing for an isoform. The default value of the missing peak probability is $\frac{n}{N}$, where $N$ is the number of total theoretical sound peaks in the HomMTM spectra for all of the reported HomMTM PSMs, $n$ is the number of the total missing peaks. The remaining masses are added to $S^{*}_{1}$. $S^{*}_{2}$ is generated in the same way, which contains some masses of the peaks corresponding to the theoretical ions of the amino acids in $F_{2}$. Then, the masses in $S^{*}_{1}$ and $S^{*}_{2}$ are used to generate the sound peaks in $S^{\prime}$ as follows:

- Step 1: for each mass $m$ in $S^{*}_{1}$, a sound peak $p$ is added to $S^{\prime}$, where the mass of $p$ is $m$, and the intensity $q$ of $p$ is computed as
  (1) \begin{align*}& q = \left\{ \begin{array}{@{}l} (q^{\prime}_{1} + q^{\prime}_{2})(1+e), m\in S^{*}_{2}\\ q^{\prime}_{1}(1+e), otherwise \\ \end{array} \right.\end{align*}
- Step 2: for each mass $m$ in $S^{*}_{2}$, if $m \notin S^{*}_{1}$, a sound peak $p$ is added to $S^{\prime}$, where the mass of $p$ is $m$, and the intensity of $p$ is $q = q^{\prime}_{2}(1+e)$,

where $q^{\prime}_{1}$ and $q^{\prime}_{2}$ are the theoretical intensities of the peaks corresponding to $F_{1}$ and $F_{2}$, respectively. Let $a_{1}$ and $a_{2}$ be the desired abundances corresponding to $F_{1}$ and $F_{2}$ in $S^{\prime}$, respectively, we have $q^{\prime}_{1}= \frac{a_{1}(q_{1}+q_{2})}{a_{1}+a_{2}}$ and $q^{\prime}_{2} = \frac{a_{2}(q_{1}+q_{2})}{a_{1}+a_{2}}$. $e$ is a random relative error that follows the Gaussian distribution $G(\mu , \sigma ^{2})$, where $\mu $ and $\sigma ^{2}$ are the mean and variance of the relative errors of intensities of all of the sound peaks in the real HomMTM spectra for the 811 HomMTM PSMs obtained from U2OS dataset and the 3800 HomMTM PSMs obtained from UCEC dataset.

*Generating noise peaks* After adding the sound peaks to $S^{\prime}$, we need to generate noise peaks. In order to make the simulated spectrum as similar as possible to the real spectrum, the simulated noise peaks are generated based on the statistics of the noise peaks in $S$. The number of noise peaks we generate for $S^{\prime}$ is the same as the number of noise peaks in $S$. Let $M_{prec}$ be the precursor mass of $S$, the masses of the generated noise peaks are random values following the Gaussian distribution $G(\mu _{mass},\sigma ^{2}_{mass})$, where $\mu _{mass} = \frac{M_{prec}}{2}, \sigma _{mass} = \frac{\mu }{2}$. Similarly, the intensities of the generated noise peaks are random values that follow the Gaussian distribution $G(\mu _{inten},\sigma ^{2}_{inten})$, where $\mu _{inten}$ and $\sigma ^{2}_{inten}$ are the mean and variance of the intensities of the noise peaks in $S$.

**Simulated HomMTM dataset:** A total of 50 simulated HomMTM PSM datasets are constructed for the evaluation of the isoforms identification performance of our method, where the proportions of abundances of two isoforms for the simulated HomMTM PSMs in different datasets vary from $1:99$ to $50:50$. Each dataset contains 500 simulated HomMTM PSMs.

To construct a simulated dataset containing 500 simulated HomMTM PSMs, we randomly select 250 real HomMTM PSMs from the 811 HomMTM PSMs obtained from U2OS dataset and 250 real HomMTM PSMs from the 3800 HomMTM PSMs obtained from UCEC dataset. For each of the 500 real HomMTM PSM, a simulated HomMTM spectrum is generated, combining the original peptide sequence to form a simulated HomMTM PSM. Note that the two isoforms reported for each real HomMTM PSM in our previous experiment (See subsection *Real Datasets*.) are regarded as the ground-truth isoforms of the corresponding simulated HomMTM PSM.

**Figure 8:**
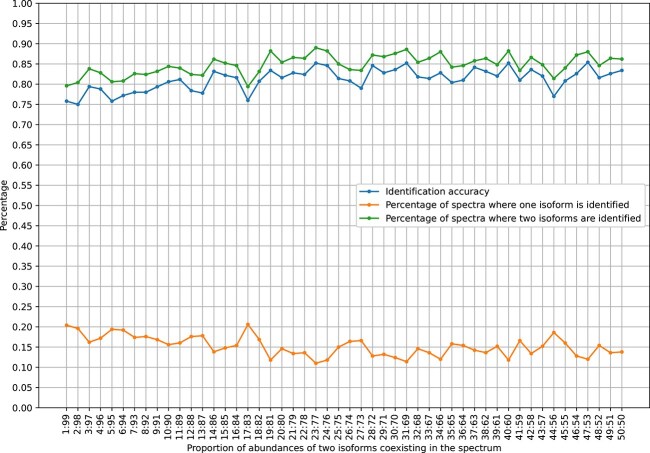
Coexisting isoforms identification results on various simulated datasets.

We use our method to conduct the isoforms identification and quantification for the simulated HomMTM PSMs in the 50 simulated datasets. The identification accuracies of different datasets are depicted by the blue dots in Fig. 8, representing the percentage of the simulated HomMTM PSMs where the two ground-truth isoforms are correctly identified. The green dots in Fig. 8 represent the percentages of spectra where two different isoforms are identified, while the orange dots represent the percentages of spectra where only one isoform is identified. The average identification accuracy is $81.2\%$. The highest identification accuracy is $85.4\%$, corresponding to the dataset where the proportion of abundances of two isoforms is $47:53$, while the lowest identification accuracy is $75\%$, corresponding to the dataset where the proportion of abundances of two isoforms is $2:98$.

According to the results, although the identification accuracies for different datasets do not differ significantly, the large difference between the abundances of two isoforms affects the performance of our method to some extent. The identification accuracies are below $80\%$ for the datasets where the proportions of two isoforms are lower than $10:90$. However, for the rest of the datasets, the identification accuracies rarely drop below $80\%$. Besides, the percentages of spectra where only one isoform is identified are higher than $15\%$ for the datasets where the proportions of two isoforms are lower than $10:90$, which occur less often for the rest of the datasets.

## Conclusion

We proposed an algorithm for phosphopeptide coexisting isoforms identification with peak error corrections based on bottom-up tandem mass spectrum and produced a program package in C++. Our experimental results on real-world data demonstrate the capability to identify isoform combinations with at most two different isoforms. Additionally, experiments on simulated datasets show satisfactory performance in isoform identification. This is a small step for the identification of coexisting peptide isoforms, which is very much needed in the field of bottom-up MS-based proteomics. Still, it remains an intriguing challenge to design algorithms for identifying and quantifying mixture spectra with a larger number of isoforms. The source code of our algorithm is available at https://github.com/KunyiE1/CoexistingModsIQ-master.

**Key Points**

- The existing peptide identification tools report only one peptide isoform for a given query spectrum, ignoring the probability that multiple isoforms coexist in one single spectrum, which is important for analyzing how different combinations of modifications coexist and compete in cells, leading to diverse functional outcomes
- We present a workflow to find coexisting combinations of PTMs, aiming at exploring the possibility of simultaneously identifying multiple isoforms from the query spectrum.
- We develop a simulator for generating simulated spectrum with the corresponding ground-truth isoforms and their abundances.
- Our experimental results on real-world data demonstrate the capability to identify isoform combinations with at most two different isoforms. Additionally, experiments on simulated datasets show satisfactory performance in isoform identification.

## Supplementary Material

Supplementary_Information_bbaf653

## Data Availability

The U2OS dataset can be obtained from the PRIDE database with the accession key PXD023665 [21]. The UCEC dataset can be obtained from Proteomic Data Commons (PDC) with the accession key PDC000126 [22]. The enriched phosphoproteomics dataset can be obtained from the PRIDE database with the accession key PXD041271 [25]. The simulated dataset is available at https://github.com/KunyiE1/CoexistingModsIQ-master. The human protein database can be obtained from UnitProt with Proteome ID UP000005640.
