# Supplementary material for: Finding coexisting combinations of posttranslational modifications with HomMTM spectra
Source: Brief Bioinform. 2025 Dec 5;26(6):bbaf653. doi: 10.1093/bib/bbaf653 (PMC13223601; doi:10.1093/bib/bbaf653)
Supplement: Supplementary_Information_bbaf653 [file supplementary_information_bbaf653.pdf]

Supplementary Information for: Finding Coexisting Combinations of Post-translational Modifications with HomMTM spectra

Kunyi Li[1] and Lusheng Wang[1,2*]

[1*]Department of Computer Science, City University of Hong Kong, 83 Tat Chee Ave, Hong Kong, China.
[2]City University of Hong Kong Shenzhen Research Institution, Shenzhen, China.

*Corresponding author(s). E-mail(s): cswangl@cityu.edu.hk;
Contributing authors: kunyili3-c@my.cityu.edu.hk;

1 Supplementary Note: Comparison with Database Search of Chimeric Spectra

A mass spectrometer typically produces two types of spectra, including mass spectra (MS1) containing ions from intact peptides and tandem mass spectra (MS2) comprising fragmented ions from selected peptides. Data-dependent acquisition (DDA) is one of the strategies to select peptides for fragmentation. In DDA, peptide ions within narrow mass-to-charge windows (typically with ranges from 0.7 to 2.0 Th) are isolated and fragmented to generate MS2 [1]. In the traditional database search framework, the peptide identification tools search the query MS2 against the candidate peptides within a narrow mass tolerance around the precursor mass reported by the mass spectrometer, assuming that each MS2 is fragmented from one single peptide precursor. However, recent studies note that peptides with different precursor masses and charge states within the narrow isolation window could still possibly be co-fragmented, generating *chimeric* MS2 spectra [2]. Supplementary Fig. 1 shows two different database search workflows depending on whether the query spectrum is considered a chimeric spectrum. The strategies for conducting false discovery rate (FDR) control in the database search for chimeric spectra and the traditional database search are very similar. Typically, PSMs reported for the query spectra are merged together and q-values are calculated for each PSM. PSMs with low q-values are removed to control the FDR of the final identification results.

The database search of chimeric MS2 spectra aims to identify all possible co-fragmented peptide (with modifications) precursors within the isolation window in the MS1 spectrum. The multiple identified peptides (with modifications) could originate from different proteins or different places of the same protein and their corresponding precursor masses may differ. Unlike the database search of the chimeric spectra that aims to identify different peptide precursors with large differences in precursor mass depending on the charge state and m/z within the isolation window, our method focuses on identifying two isoforms with the same precursor mass, originating from the same unmodified peptide, coexisting in one MS2 spectrum. Our method is applied to the PSMs reported by the traditional database search. For each PSM, the peptide reported by the search engine and the precursor mass are parts of the input for our method. In fact, our method serves as a post-processing step after the database search under the FDR control. See Supplementary Fig. 2.

We also try to use chimeric database search tools to identify coexisting isoforms with the same precursor mass. We search the spectra in the enriched phosphoproteomics dataset (mentioned in Section "Results") against the human protein database using Sage v0.14.7 [3], following the same modification settings used by Chang et al. The chimeric search is enabled, and the number of PSMs reported for each query chimeric spectrum is up to 2. The precursor mass tolerance is 0.1 Da. The maximum number of variable modifications allowed in the peptide is two. (If the maximum number of variable modification is set to be larger than two, our computer with 64GB memory fails to allocate enough memory for Sage due to the large search space.) The results are displayed in Supplementary Table 1. Under the 1% FDR control at PSM level, 19537 PSMs are reported for 19356 spectra. Among the 19356 identified spectra, 19175 spectra are matched to one single peptide, while 181 spectra are matched to two co-fragmented peptides. Among the 181 chimeric spectra, only 42 chimeric spectra are identified to be co-fragmented from two coexisting isoforms with the same precursor mass, orignating from the same unmodified peptides. The number of chimeric spectra co-fragmented from peptides originating from different unmodified peptide is 139, which is about 3.3 times that co-fragmented from two isoforms.

We can see that Sage tends to match the chimeric spectra with two peptides originating from different unmodified peptides. Since our method focuses on the coexisting isoforms identification, our method identifies more spectra (404 spectra) co-fragmented from two isoforms based on the traditional searching results.

2 Supplementary Figures

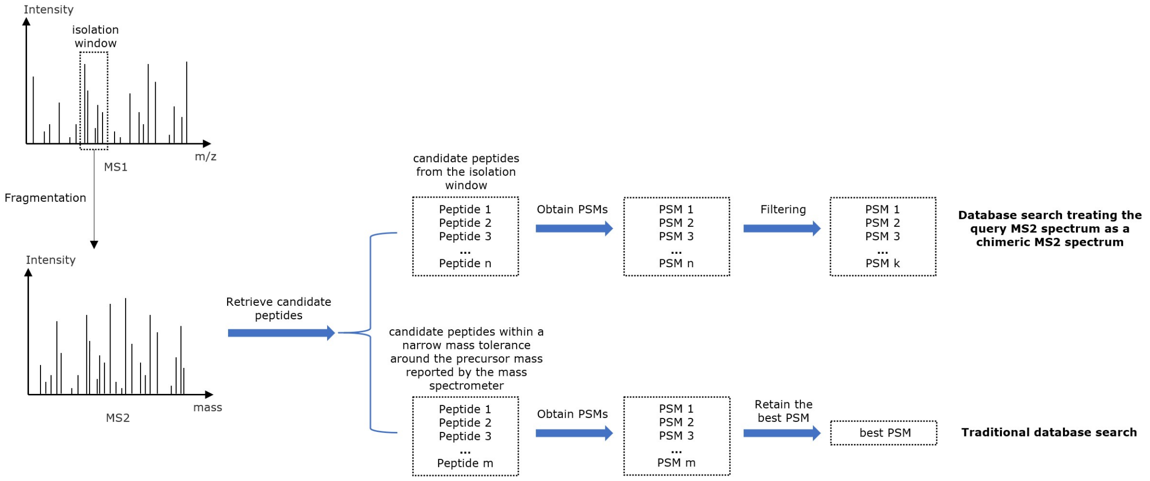

Supplementary Fig. 1 Peptide ions in the narrow isolation window are fragmented to generate the MS2. If the MS2 is considered as a chimeric spectrum, the database search tools first try to find all possible peptide precursors that could be reasonably co-fragmented within the isolation window. For each possible peptide precursor, multiple candidate peptides are retrieved from the database. Subsequently, each candidate peptide forms a peptide-spectrum match (PSM) with the MS2. The search tools calculate the scores for these PSMs and remove some low-quality PSMs based on predefined criteria. As a result, multiple PSMs are reported for one query MS2, corresponding with peptides with different precursor masses. However, in the traditional database search, the query MS2 is assumed to be fragmented from the peptide ion with a fixed precursor mass reported by the spectrometer. In this case, the MS2 is searched against candidate peptides within a narrow mass tolerance around the reported precursor mass. Each candidate peptide forms a PSM with the MS2, but only the best PSM is retained.

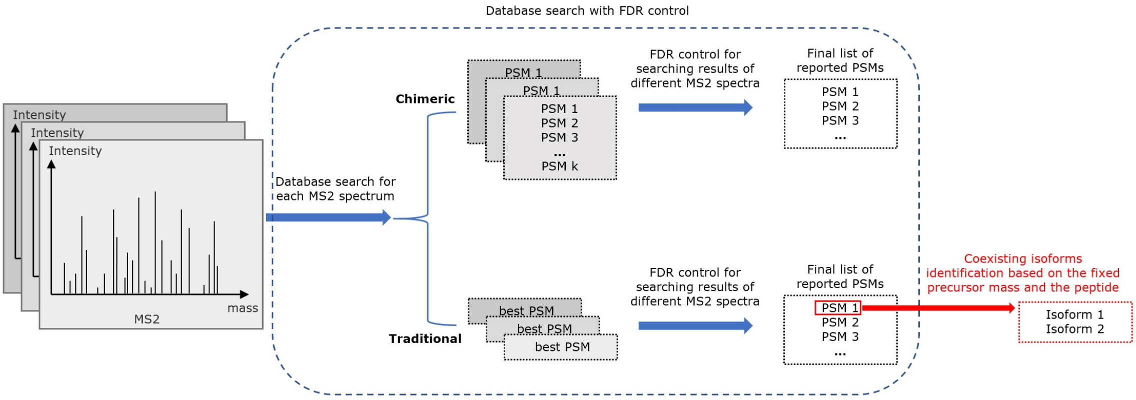

Supplementary Fig. 2 The frame work of database search with FDR control is displayed in the middle of the figure. Unlike the traditional database search, in the chimeric database search, multiple PSMs are reported for one query MS2 spectrum before being filtered to control FDR. Thus, after FDR control is conducted, there exists one or more than one PSMs reported for one query MS2. Our method serves as a post-processing step for the traditional database search results. For each PSM among the reported PSMs under the FDR control, with the fixed corresponding query spectrum, precursor mass and unmodified peptide sequence, we try to identify two coexisting isoforms.

3 Supplementary Tables

Supplementary Table 1 Searching results using Sage

Spectra Type	Number
Identified spectra	19356
Spectra matching 2 peptides	181
Spectra matching 2 isoforms of the same unmodified peptide	42

Supplementary Table 2 Definitions for key terminologies and symbols

Terms/Symbols	Definition
PMG	Peptide isoform mass graph
SMG	Spectrum mass graph
HomMTM	Homogeneous multiplexed tandem mass
HomMTM PSM	The PSM where two coexisting isoforms are identified by our method
p_j	The j-th peak in SMG
P_1 and P_2	A valid pair of paths found from the backtracking graph
$I(u)$	Intensity of the peak of node u
I_j	Intensity of peak p_j
$\varepsilon(u, v)$	Intensity error for nodes u and v which share a peak
$\varepsilon(u)_i$	Intensity error for node u which do not share the peak with any other nodes.
$S(P_1, P_2)$	Set of pairs of nodes in paths P_1 and P_2 which share a common peak
$I(P_1, P_2)$	Total peak intensity error of paths P_1 and P_2
$D(u, v)$	The minimum intensity error for the two sub-paths heading at node u and v
$f(v)$	Set of nodes v' such that there exists a edge from v' to v in the backtracking graph
$d(u, u', 1)$	Intensity error for the missing peaks from node u' to u in path P_1

References

[1] Fengchao Yu, Yamei Deng, and Alexey I Nesvizhskii. Msfragger-dda+ enhances peptide identification sensitivity with full isolation window search. *Nature Communications*, 16(1):3329, 2025.

[2] Stephane Houel, Robert Abernathy, Kutralanathan Renganathan, Karen Meyer-Arendt, Natalie G Ahn, and William M Old. Quantifying the impact of chimera ms/ms spectra on peptide identification in large-scale proteomics studies. *Journal of proteome research*, 9(8):4152–4160, 2010.

[3] Michael R Lazear. Sage: an open-source tool for fast proteomics searching and quantification at scale. *Journal of Proteome Research*, 22(11):3652–3659, 2023.
